# Liver metastasis from hepatoid adenocarcinoma of the stomach: a case report and literature review

**DOI:** 10.3389/fonc.2024.1297062

**Published:** 2024-06-27

**Authors:** Hui Zhu, Qingqing Li, Linqing Qian

**Affiliations:** Department of Radiology, Suzhou Wuzhong People’s Hospital, Suzhou, China

**Keywords:** hepatoid adenocarcinoma of the stomach, hepatic metastasis, magnetic resonance imaging, radiomics, case report

## Abstract

Hepatoid adenocarcinoma of the stomach (HAS) represents a rare malignant neoplasm sharing morphological and immunophenotypic similarities with hepatocellular carcinoma (HCC). Pathological morphology serves as the cornerstone for diagnosis, often accompanied by elevated alpha-fetoprotein (AFP) levels, nonspecific clinical symptoms, and imaging features reminiscent of gastric adenocarcinoma (GA). Liver metastases from HAS can mimic the enhancement patterns of HCC, posing challenges in differentiation from high-risk HCC cases. Conversely, HAS typically exhibits poorer prognostic outcomes compared to HCC and GA. This report presents a case of HAS with liver metastasis alongside a comprehensive literature review covering its pathology, molecular mechanisms, clinical presentations, and treatment modalities. Special focus is given to imaging characteristics and the utilization of radiomics for early-stage detection. The integration of imaging findings with laboratory results aids in HAS diagnosis, while radiomics provides novel insights for precise discrimination. In conclusion, the identification of distinct imaging markers distinguishing HAS from HCC and GA shows promise in facilitating optimal treatment strategies and improving patient outcomes.

## Background

Hepatoid adenocarcinoma of the stomach (HAS) is a rare malignant neoplasm, characterized by histological and immunophenotypic resemblance to hepatocellular carcinoma (HCC). This malignancy often presents with elevated levels of alpha-fetoprotein (AFP) and is diagnosed through pathological assessment involving morphological and immunohistochemical markers, including AFP, GPC-3, SALL4, and Hep-Par 1 (1). The incidence of HAS is limited, accounting for merely 0.17%–15% of all gastric cancers (2), with an estimated annual occurrence of 0.58–0.83 cases per million individuals (3). In 1970, Bourreill (4) first documented a case of gastric adenocarcinoma (GA) associated with heightened AFP levels. Subsequently, Ishikura (5) reported an instance of primary GA characterized by markedly elevated AFP levels (12,000 ng/ml), coining the term HAS. Subsequent investigations established that HAS may also manifest without elevated AFP levels (6), suggesting that elevated serum AFP serves as a hallmark of HAS but lacks absolute specificity. HAS is distinguished by its aggressive behavior and dismal prognosis in comparison to GA and HCC (3, 7–9), with median survival ranging from 10 months to 18 months (10, 11). Diagnosis often occurs at advanced stages, given the non-specific nature of clinical symptoms and imaging features. The latter closely resemble those of GA, and instances where liver metastases from HAS mimic HCC enhancements further complicate differentiation, especially among high-risk HCC patients. Recognition of distinct imaging markers distinguishing HAS from HCC and GA holds the potential to inform tailored therapeutic decisions and enhance patient prognoses.

## Case presentation

An 80-year-old man was admitted to our hospital due to liver tumors and abdominal distention persisting for over 4 months. A CT scan of the abdomen from 4 months ago revealed “multiple liver tumors.” Lab test results from the same time showed AFP levels of 1,212.83 ng/ml and carcinoembryonic antigen (CEA) levels of 132.53 ng/ml. At that time, the patient received only symptomatic treatment. Four months later, the patient’s upper abdominal discomfort worsened, and AFP and CEA levels were measured at 22,195.00 ng/ml and 2,964.00 ng/ml, respectively. The patient had a medical history of hypertension, coronary heart disease, atrial fibrillation, and cardiac insufficiency for over 20 years. There was no history of hepatitis or family history of liver cancer.

**Figure 1:**
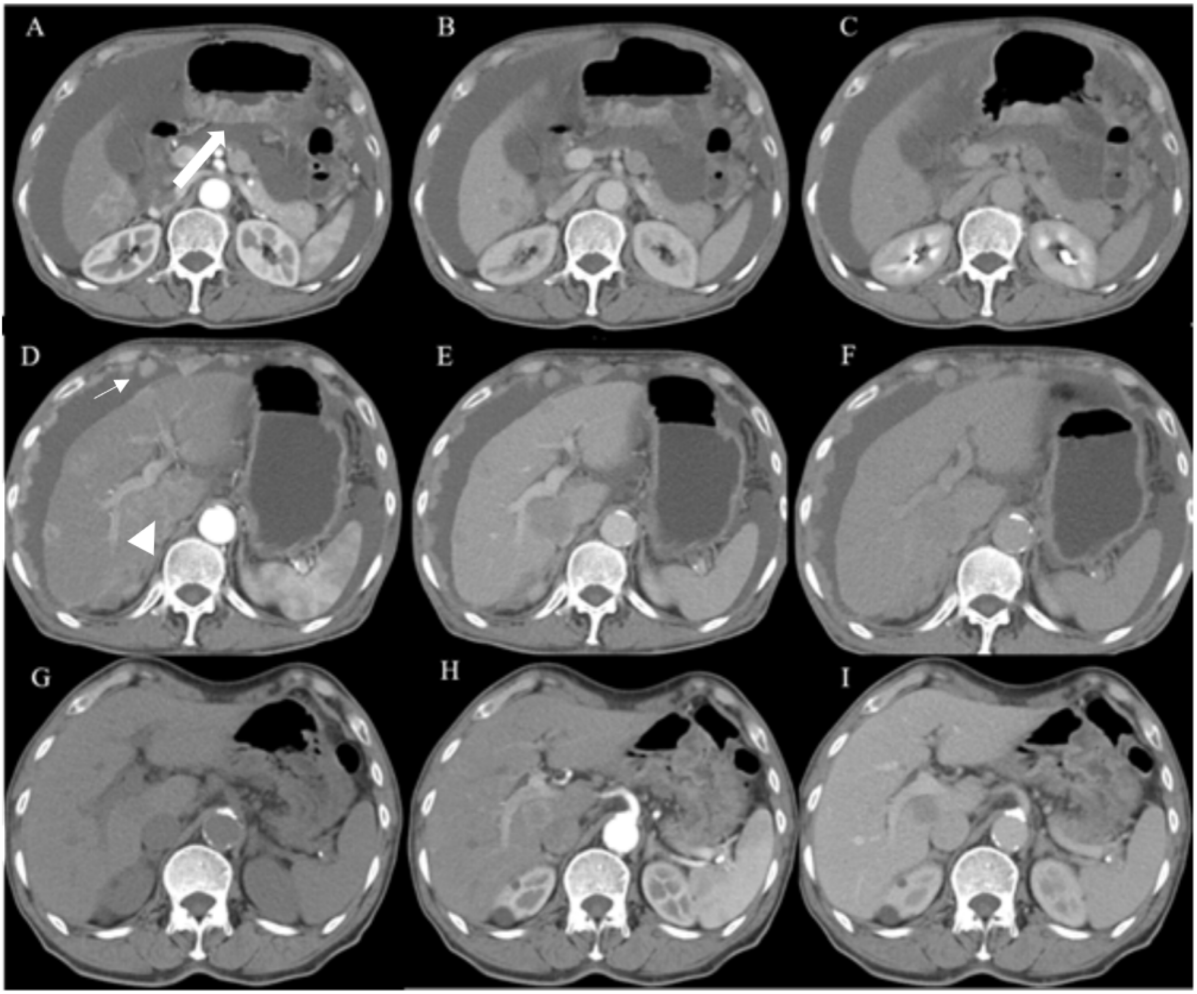
Axial enhanced CT images in a patient with HAS. **(A–C)** The CT scan revealed thickening of the gastric wall (arrow), with enhanced appearance in the gastric sinus area. The CT values of the lesion were 34 HU, 76 HU, and 54 HU in the unenhanced, arterial, and portal phases, respectively. **(D–F)** Multiple circular intensified lesions and nodes were observed in the peritoneum (straight arrow), along with heterogeneous enhancement in the retroperitoneal lymph nodes. Abundant fluid was seen in the abdominopelvic cavity. The lesion in the S5 liver segment exhibited marked heterogeneous enhancement (triangle). **(G–I)** The patient’s CT images from 4 months earlier displayed multiple enhanced lesions encircling the liver during the arterial phase.

**Figure 2:**
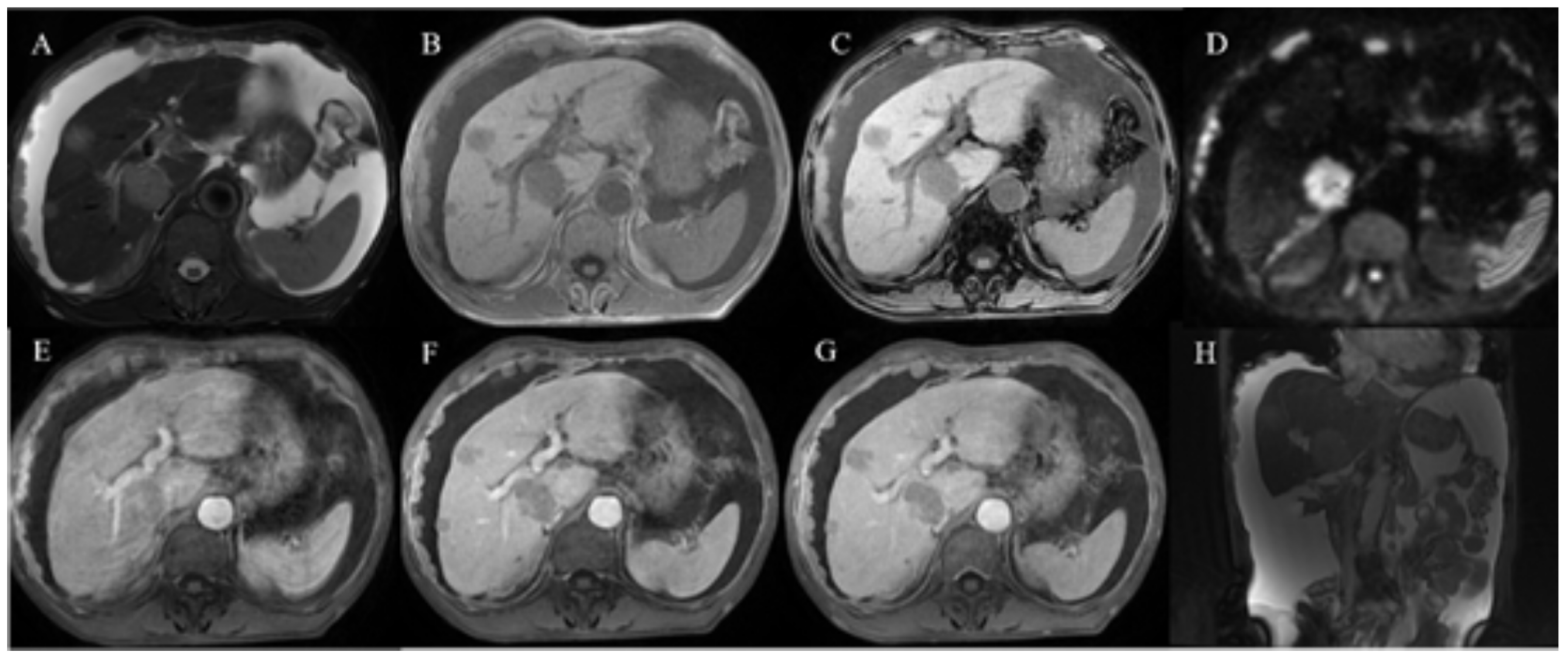
Abdominal enhanced MR images in a patient with HAS. **(A–H)** Multiple nodular and mass-like abnormal lesions were detected in the liver. Some of these lesions appeared fused together with indistinct boundaries. They exhibited hyperintensity on T2WI images **(A)** and hypointensity on T1WI images **(B, C)**. Notably, there was no substantial reduction in lesion intensity after fat saturation, indicating that the lesions lacked fat content. Significant diffusion restriction was also observed **(D)**. The lesions displayed progressive enhancement **(E–G)**, with the liver parenchyma demonstrating low signal intensity. Moreover, the small omental capsule, hepatoportal, and multiple retroperitoneal lymph nodes showed heterogeneous enhancement, and the reticulum displayed knot-like thickening with varying enhancement.

**Figure 3:**
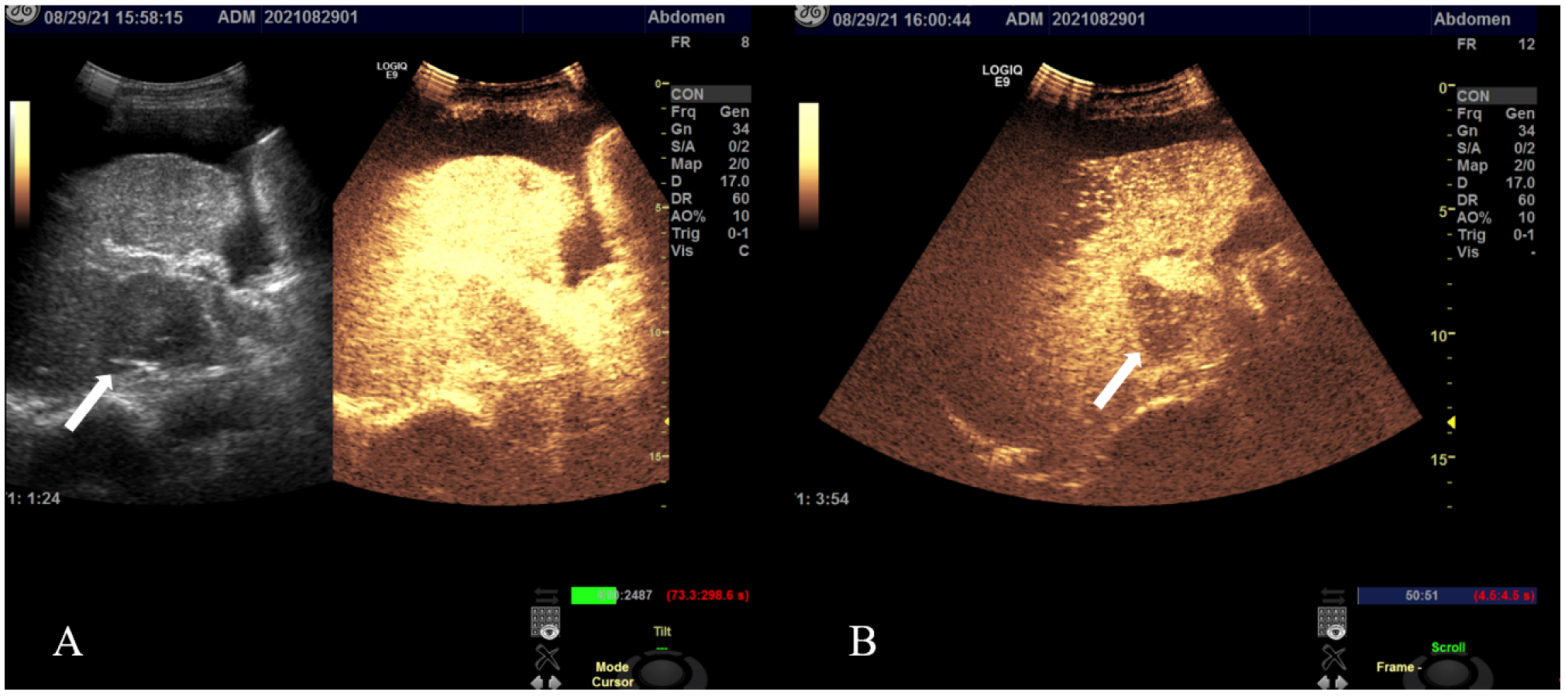
Contrast-enhanced ultrasound images in a patient with HAS: multiple regions exhibited echogenic enhancement during the late arterial phase, followed by attenuation in the portal phase and continued attenuation in the delayed phase. In the S5 liver segment, a mass (arrow) demonstrated a fast-in-fast-out pattern **(A)**, remaining hypo-enhanced even after 3 min **(B)**.

**Figure 4:**
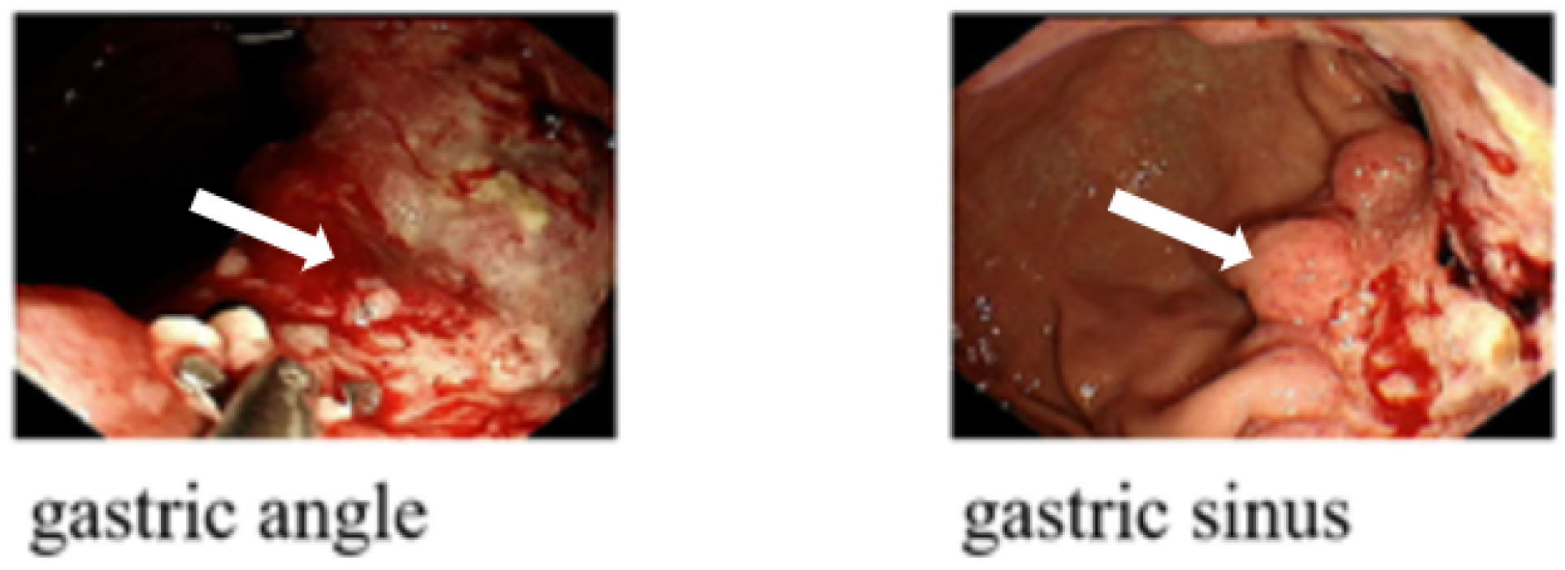
Gastroscopy images in a patient with HAS: the gastroscopic examination revealed infiltrative hyperplastic foci located in the gastric sinus and gastric angle (arrow).

A contrast-enhanced CT scan (**Figure 1**) was conducted using a 128-row CT scanner (Siemens SOMATOM Definition AS). The images revealed gastric wall thickening with enhancement in the gastric sinus area. Lesion CT values were 34 Hounsfield Units (HU) in the unenhanced phase, 76 HU in the arterial phase, and 54 HU in the portal phase post-contrast injection. Cirrhotic changes were evident. Multiple circular intensified lesions and nodes were noted in the peritoneum, with heterogeneous enhancement in retroperitoneal lymph nodes. A significant amount of fluid was present in the abdominopelvic cavity. The S5 liver segment lesion exhibited marked heterogeneous enhancement. Upper abdominal enhanced magnetic resonance imaging (MRI) (**Figure 2**) was performed using a 1.5-T system (GE Signa HDe), revealing multiple nodular and mass-like abnormal lesions in the liver. Some lesions appeared fused with unclear boundaries. Lesions showed hyperintensity on T2WI images and hypointensity on T1WI images. Fat saturation did not significantly reduce lesion intensity, suggesting fat-free lesions. Significant diffusion restriction was also observed. Contrast-enhanced ultrasound (**Figure 3**) demonstrated multiple areas of increased echogenicity during the late arterial phase, with attenuation in the portal phase and further attenuation in the delayed phase. In the S5 segment, a mass with a rapid-in-rapid-out enhancement pattern was observed, remaining hypo-enhanced after 3 min. Gastroscopy (**Figure 4**) revealed areas of infiltrative tissue growth in the gastric sinus and gastric angle. Biopsy of the gastric sinus and horn (**Supplementary Figure S1**) indicated moderately differentiated adenocarcinoma, confirmed as HAS through immunohistochemistry. Staining for CK7, AFP, Her-2, PMS2, MLH1, MSH6, MSH2, and Ki-67 was positive, with a Ki-67 value of 80%. The patient tolerated Tegio chemotherapy well during hospitalization, with intermittent abdominal drainage and albumin infusion to relieve abdominal swelling. The patient was discharged after half a month of relief of abdominal distension symptoms, but the patient’s prognosis was poor, and telephone follow-up results showed that the patient died.

## Discussion and conclusion

HAS, an aggressive subtype of gastric cancer, is characterized by regions with differentiation resembling hepatocellular features. Several case series and retrospective studies have investigated pathological characteristics, molecular mechanisms, treatment approaches, and prognosis related to HAS (12). Despite these efforts, the precise causes of HAS and the potential involvement of the AFP gene in its development remain uncertain. Some researchers propose that HAS might arise from pluripotent stem cells, and mutations in genes like TP53 and ATBF1 could contribute to AFP production in gastric cancer cells (12, 13). Patients with HAS often exhibit nonspecific symptoms including abdominal pain, bloating, and discomfort. While treatment strategies for HAS remain a topic of debate, certain studies have yielded promising outcomes.

Accurate diagnosis of HAS relies on the expression of various tumor markers. Multiple investigations have established a significant association between AFP levels and patient prognosis (14). A retrospective study indicated that elevated AFP levels independently correlated with a poorer prognosis. In instances where AFP measurement is unavailable, carbohydrate antigen 19–9 (CA 19–9) could serve as a reasonably effective alternative for predicting 3-year overall survival (OS) and recurrence-free survival (RFS) among HAS patients (15). An optimal AFP threshold for 3-year OS and RFS was determined to be 516 ng/ml. Notably, research findings (15, 16) underscore AFP’s pivotal role in prognosticating HAS outcomes. Moreover, heightened AFP levels indicate an increased likelihood of liver metastasis. In our case, the patient’s initial AFP and CEA levels were 1,212.83 ng/ml and 132.53 ng/ml, respectively, coinciding with liver metastases. Subsequently, the patient’s AFP surged to 22,195.00 ng/ml, CEA escalated to 2,964.00 ng/ml, and the extent of liver metastases notably expanded, aligning with the aforementioned perspective.

Various imaging modalities, including CT, MRI, and positron emission tomography/CT (PET-CT), provide valuable tools for distinguishing HAS from other conditions. Scholars in recent years focused on correlating imaging features with HAS pathology to enhance understanding, enable early diagnosis, and improve prognostication for patients (17, 18). On CT scans, HAS cases frequently exhibited ulceration, thickened gastric walls, and persistent enhancement, similar to features seen in GA. Infiltration into extracapsular fat indicated heightened aggressiveness (19, 20). Recent research highlighted that compared to non-HAS cases, HAS exhibited higher plain CT attenuation and arterial phase CT attenuation values, alongside a reduced mean CT attenuation difference between arterial and venous phases (18). Comparing HAS to GA, HAS displayed higher serum AFP and CEA levels, greater enhancement in the portal venous phase, larger mean short diameter of metastatic lymph nodes (MSD), longest short diameter of metastatic lymph nodes on CT (LSD), and a higher ratio of enlarged lymph nodes on CT to metastatic lymph nodes pathologically (ELN/MLN). This suggests that HAS was characterized by pronounced arterial phase enhancement, while GA exhibited gradual enhancement (21). Imaging features of HAS combined with liver metastases exhibited considerable variability. Abundant blood supply to HAS liver metastases rendered them clearly visible during the arterial phase and recognizable during the venous and delayed phases. This similarity to characteristics of HCC posed difficulties in differentiation, particularly in high-risk HCC patients (22, 23). In this instance, the S5 liver lesion displayed “fast in and out” enhancement in ultrasonography, complicating the distinction between HCC and HAS with hepatic metastasis. Some scholars proposed that hypo-intensity during the arterial phase was more common in HAS liver metastases than in HCC. Imaging indicators for HAS included isolated tumor thrombosis in the portal vein and tumor necrosis in hepatic nodules. Notably, even nodules smaller than 1 cm in diameter exhibited frequent necrosis in HAS liver metastases, a phenomenon less prevalent in HCC (10%–40%) (18). MRI effectively detected necrosis, thereby aiding in HAS diagnosis. Moreover, HAS was less likely to create fibrous-like pseudocysts due to its rapid progression, distinguishing HAS with intrahepatic metastases from HCC. In this case, MRI indicated slight enhancement during the hepatic S5 lesion’s arterial phase and an absence of pseudocyst formation, bolstering the diagnosis of liver metastasis from HAS. Scholars also pursued the identification of HAS and liver metastasis using PET/CT. Xu et al. reported a rise in the SUVmax value to 13.6 in the gastric mass and lymph node metastasis of HAS, indicating intense FDG uptake and reflecting severe malignancy. Other studies similarly underscored the importance of PET/CT in precise diagnosis and staging of HAS (24–26).

The advancement of medical imaging technology and quantitative analysis have led to the emergence of radiomics, a superior method of quantitative image analysis compared to traditional qualitative or semi-quantitative methods. Studies focusing on developing nomograms to predict recurrence and prognosis in gastric cancer and gastric endocrine tumors have demonstrated the significant benefits of combining imaging metrics for disease differentiation and prognosis assessment (27–29). Some researchers have utilized CT-based radiomics nomograms to accurately distinguish between individuals with HAS and those with gastric adenocarcinoma. Wang et al. (30) extracted 1,409 features and derived a radscore comprising 19 features. They observed that the CT-based nomogram, including radscore, AFP, and CA 724, effectively discriminated between HAS and GA. Lin et al. (31) formulated a recurrence-free survival (RFS) nomogram incorporating independent prognostic factors such as age, preoperative CEA, number of examined lymph nodes, perineural infiltration, and lymph node ratio. This nomogram proved valuable, complementing the American Joint Committee on Cancer TNM staging system. Currently, there is a lack of reports on the application of advanced sequences such as arterial spin labeling (ASL), perfusion-weighted imaging (PWI), and intravoxel incoherent motion imaging (IVIM) in HAS perfusion. Further research is necessary to evaluate radiology’s efficacy in preoperative identification, staging, and prognosis determination for HAS and HCC.

Hepatoid adenocarcinoma of the stomach (HAS) represents a rare and aggressive neoplasm with a grim prognosis. Typically, it manifests with elevated alpha-fetoprotein (AFP) levels, nonspecific clinical symptoms, and imaging features resembling benign gastric adenomas (GA). Moreover, liver metastases from HAS may present similar enhancement patterns to high-risk hepatocellular carcinoma (HCC), complicating accurate differentiation. Recent advancements in understanding HAS’s imaging characteristics have emerged. The integration of imaging findings with laboratory results can facilitate the diagnosis of HAS. The implementation of radiomics has provided novel insights for the precise differential diagnosis of HAS.

## Data availability statement

The original contributions presented in the study are included in the article/**Supplementary Material**. Further inquiries can be directed to the corresponding author.

## Ethics statement

The studies involving humans were approved by Suzhou Wuzhong People’s Hospital Ethics Committee [2023-ky-037]. The studies were conducted in accordance with the local legislation and institutional requirements. The participants provided their written informed consent to participate in this study. Written informed consent was obtained from the individual(s) for the publication of any potentially identifiable images or data included in this article.

## Author contributions

HZ: Writing – original draft. QL: Writing – original draft. LQ: Writing – original draft.

## Supplementary material

Supplementary Figure 1 Biopsy Pathological Images of Patients with Hepatic Adenocarcinoma (HAS): **(A)** HE Staining, 100x Magnification: The tumor displayed polygonal cell cords characterized by prominent nuclei and nucleoli. Some cells exhibited ample eosinophilic or transparent cytoplasm, indicating hepatocytoid differentiation. **(B)** AFP Immunohistochemical Staining, 100x Magnification: AFP expression was primarily detected within the cytoplasm of tumor cells situated in the region demonstrating hepatocellular differentiation.
