# Supplementary figures and images for: Liver metastasis from hepatoid adenocarcinoma of the stomach: a case report and literature review
Source: Front Oncol. 2024 Jun 27;14:1297062. doi: 10.3389/fonc.2024.1297062 (PMC11236608; doi:10.3389/fonc.2024.1297062)

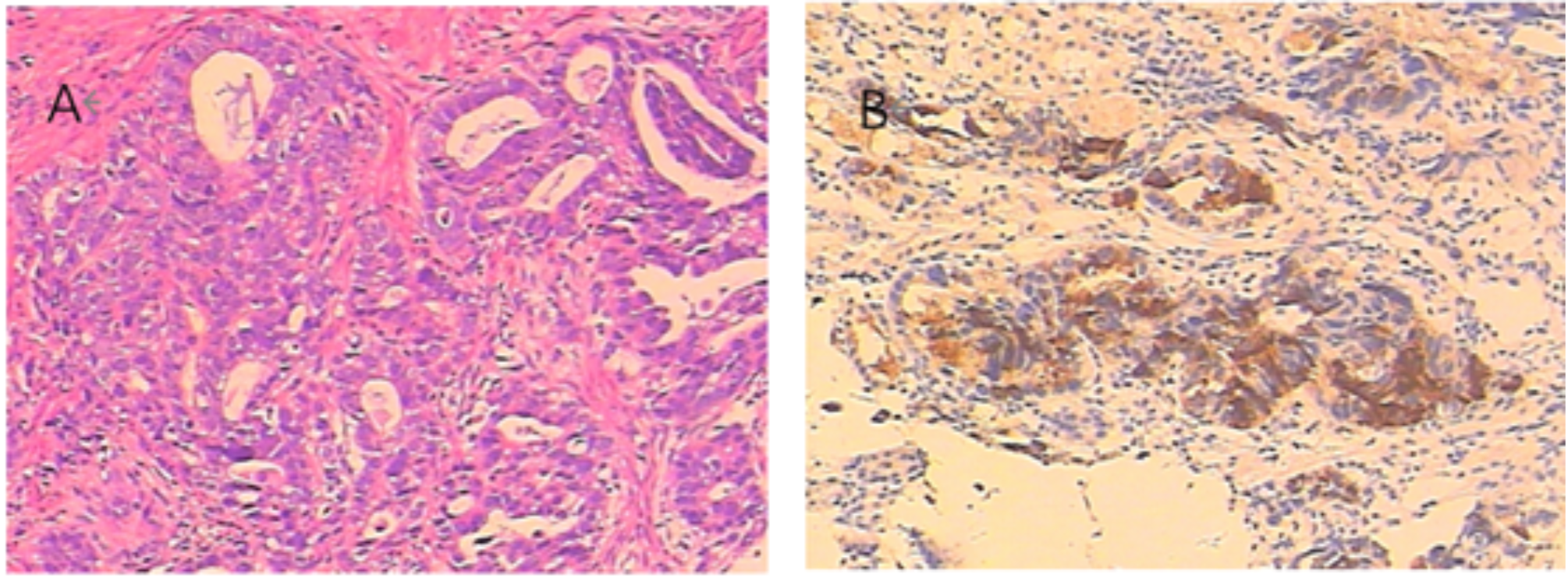

Supplement: Supplementary Figure 1 — Biopsy Pathological Images of Patients with Hepatic Adenocarcinoma (HAS): (A) HE Staining, 100x Magnification: The tumor displayed polygonal cell cords characterized by prominent nuclei and nucleoli. Some cells exhibited ample eosinophilic or transparent cytoplasm, indicating hepatocytoid differentiation. (B) AFP Immunohistochemical Staining, 100x Magnification: AFP expression was primarily detected within the cytoplasm of tumor cells situated in the region demonstrating hepatocellular differentiation. [file Image_1.tif]
